# Antitumor Effects of Intra-Arterial Delivery of Albumin-Doxorubicin Nanoparticle Conjugated Microbubbles Combined with Ultrasound-Targeted Microbubble Activation on VX2 Rabbit Liver Tumors

**DOI:** 10.3390/cancers11040581

**Published:** 2019-04-24

**Authors:** Jae Hwan Lee, Hyungwon Moon, Hyounkoo Han, In Joon Lee, Doyeon Kim, Hak Jong Lee, Shin-Woo Ha, Hyuncheol Kim, Jin Wook Chung

**Affiliations:** 1 Department of Radiology, Seoul National University Bundang Hospital, 82 Gumi-ro 173, Bundang-gu, Seongnam 13620, Korea; lzhwanmd@gmail.com (J.H.L.); moondaeng82@naver.com (H.M.); hakjlee@gmail.com (H.J.L.); 2 Department of Chemical & Biomolecular Engineering, Sogang University, 35 Baekbeom-ro, Mapo-gu, Seoul 04107, Korea; 507513@hanmail.net (H.H.); doyeon777@naver.com (D.K.); 3 Department of Radiology, National Cancer Center, 323 Ilsan-ro, Ilsandong-gu, Goyang 10408, Korea; cheolh@gmail.com; 4 Department of Radiology, Seoul National University College of Medicine, Daehak-ro, Jongno-gu, Seoul 03080, Korea; 5 IMGT Co., Ltd., 172 Dolma-ro, Bundang-gu, Seongnam 13605, Korea; shinwoo.ha@gmail.com; 6 Institute of Radiation Medicine, Seoul National University Hospital, 101, Daehak-ro, Jongno-gu, Seoul 03080, Korea

**Keywords:** albumin nanoparticles, microbubble, ultrasound, theranostics, hepatocellular carcinoma, VX2 tumor, intra-arterial chemotherapy

## Abstract

Image-guided intra-arterial therapies play a key role in the management of hepatic malignancies. However, limited clinical outcomes suggest the need for new multifunctional drug delivery systems to enhance local drug concentration while reducing systemic adverse reactions. Therefore, we developed the albumin-doxorubicin nanoparticle conjugated microbubble (ADMB) to enhance therapeutic efficiency by sonoporation under exposure to ultrasound. ADMB demonstrated a size distribution of 2.33 ± 1.34 µm and a doxorubicin loading efficiency of 82.7%. The echogenicity of ADMBs was sufficiently generated in the 2–9 MHz frequency range and cavitation depended on the strength of the irradiating ultrasound. In the VX2 rabbit tumor model, ADMB enhanced the therapeutic efficiency under ultrasound exposure, compared to free doxorubicin. The intra-arterial administration of ADMBs sufficiently reduced tumor growth by five times, compared to the control group. Changes in the ADC values and viable tumor fraction supported the fact that the antitumor effect of ADMBs were enhanced by evidence of necrosis ratio (over 70%) and survival tumor cell fraction (20%). Liver toxicity was comparable to that of conventional therapies. In conclusion, this study shows that tumor suppression can be sufficiently maximized by combining ultrasound exposure with intra-arterial ADMB administration.

## 1. Introduction

Image-guided intra-arterial (IA) therapies, such as hepatic arterial infusion chemotherapy (HAIC) or trans-arterial chemoembolization (TACE) are frequently used for the treatment of primary or secondary liver cancers [1,2,3,4,5,6]. HAIC involves the local and targeted delivery of high concentrations of chemotherapeutic drugs directly to the tumor, whereas TACE, with or without drug-eluting beads, combines local and targeted drug delivery with concurrent tumor-feeding artery embolization. The theory behind this treatment recommends delivering the maximal dose of the chemotherapeutic agent to the target tissue while minimizing systemic toxicity. However, clinical response is still unsatisfactory, as local tumor control rates achieved following TACE is only 15–60%, and increases median survival without treatment from 16 months to 20 months [7,8]. One possible reason for these poor results is perhaps the limited delivery of the drug to the target tumor [9,10,11]. Changes in tumor microenvironment including decreased pH, hypoxia, and abnormal vascularity impede drug delivery to the target. Moreover, local tumor recurrences at the periphery of the treated area are common and are often a cause for treatment failures [12]. Another limitation in clinical field is side effects. Concentrations of drugs in the bloodstream are increased even though drugs are administered via IA injection. Therefore, a decrease of the drug concentration in the bloodstream is also necessary to reduce the side effects caused by target delivery of drugs. The simultaneous monitoring of drug delivery is an unmet clinical need for enhancing tumor control. Thus, novel drug delivery carriers for IA chemotherapy are mandatory for better drug delivery and to allow multimodal imaging which enables the carriers to be simultaneously visualized with various imaging modalities.

**Figure 1:**
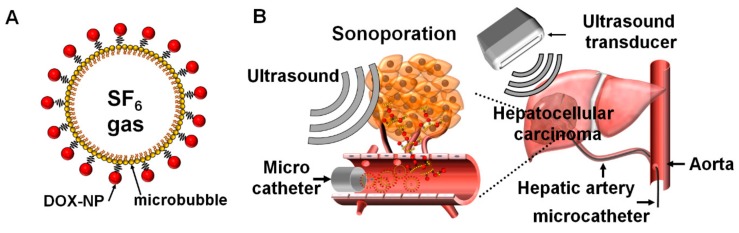
Schematic illustrations of (**A**) the ADMB complex and (**B**) treatment procedure for the intra-arterial administration of the ADMB complex using microcatheters under ultrasound exposure.

In the last decade, studies have used ultrasound as an external trigger for enhancing local drug penetration through artificial pore formation in the cell membrane. This phenomenon is known as the sonoporation effect [13,14]. Theoretically, sonoporation is based on the cavitation between ultrasound and microbubbles. Microbubbles are made to repetitively expand and shrink under ultrasound irradiation. This behavior of microbubbles induces a microstream in the blood vessel and continually puts the cellular membrane under stress (stable cavitation). The microbubbles upon extreme oscillations finally explode at the critical elastic point under the strong ultrasound intensity. At the moment of microbubble explosion, microjets and shock waves are generated and temporally drill the cell membrane near the microbubble with pores of 100–300 nm in size (inertial cavitation) [15]. This cavitation approach is utilized for enhancing local drug delivery to the target site and for improving the intracellular uptake of large molecules and non-permeable drugs. Several groups have shown that sonoporation can enhance the therapeutic efficiency of chemotherapy and gene therapy [16,17]. These researches successfully demonstrated that microbubble-encapsulated drugs induce the well-penetration to target site and functionalization for therapeutic effect. However, the use of microbubbles as drug carriers has been limited since a small portion of drug encapsulation is possible structurally, and the undesirable release of drugs by degradation in the blood stream. To overcome these limitations, nanoparticles are studied for increasing drug loading and protection. In our previous study, human serum albumin nanoparticles (HSA-NPs) were effectively delivered to the tumor site by sonoporation [18,19]. Microbubble-conjugated anticancer drug-loaded HSA-NPs enhanced the selective delivery of drugs to the tumor and led to the improvement of therapeutic efficiency, compared to the administration of pure drugs and administration without ultrasound irradiation. Nanoparticle-based drug delivery has been advantageous for improving drug loading efficiency, protection of drugs from degradation, and intracellular penetration via characteristics of the nanoparticles which allow a sustained drug release. These advantages have progressed tumor-selective delivery and functional release of the drug to the target site. However, the systemic circulation of all agents including nanoparticles is biologically limited for clearance by accumulation in the liver or kidney rather than in the tumor region [20]. Therefore, the therapeutic efficiency is not maximized for tumor treatment owing to drug cleavage. Thus, some of microbubble during the circulation are ruptured by diffusion of gas in the core part. Sequentially, the sonoporation effect should be decreased at the tumor site, compared to direct administration such as IA injection. Therefore, a more effective administration route for maximizing therapeutic efficiency would be tumor vessels rather than the systemic circulation. Unfortunately, there are few or no studies that compare the therapeutic efficiency of systemic administration to that of specific administration routes, which is a surgical procedure performed at present on humans in the clinic. In the case of hepatocellular carcinoma, the IA administration of drugs using microcatheters has been intensively applied in clinical practice, for the local administration of anticancer drugs and chemotherapeutic embolic agents [1,3,4]. The purpose of our study was to explore the antitumor effect of ADMBs combined with ultrasound-targeted microbubble activation (ADMB/US) in rabbit VX2 liver tumor model. (Figure 1A) and to compare antitumor effect via IA administration to IV administration. (Figure 1B).

## 2. Results

### 2.1. Preparation of the ADMB Complex

**Figure 2:**
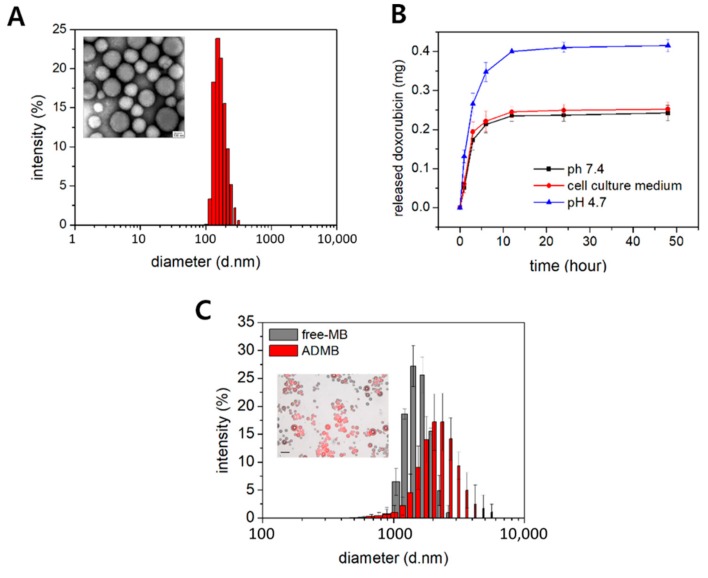
Characteristics of the albumin-doxorubicin nanoparticle and the ADMB complex (**A**) size distribution and TEM image (inset image, scale bar:100 nm) of the albumin-doxorubicin nanoparticle. (**B**) In vitro release of doxorubicin from albumin-doxorubicin nanoparticles at pH 7.4, pH 4.7 and DMEM containing 10% of fetal bovine serum and 1% of antibiotics. (**C**) size distribution of free MB (gray) and ADMB (red). Merged fluorescence and optical images (inset image, scale bar: 20 µm).

For the enhancement of therapeutic effect by sonoporation phenomenon, ADMB complex was developed by self-assembled microbubble and albumin-doxorubicin nanoparticles. Albumin-doxorubicin nanoparticles were fabricated by the dropwise addition of ethanol to albumin. A size distribution of albumin-doxorubicin nanoparticles of 205.5 ± 45.3 nm. (PDI; 0.172) was demonstrated. Transmission electron microscope images of the albumin-doxorubicin nanoparticles demonstrated a uniform and spherical morphology (Figure 2A). The loading efficiency of doxorubicin into the albumin-doxorubicin nanoparticles was 82.7%. The doxorubicin was released in a sustained manner from the albumin-doxorubicin nanoparticles at an in vitro release rate of 24.2% for 50 h with the initial burst. Thus, albumin-doxorubicin nanoparticles demonstrated a similar release profile as doxorubicin in cell culture medium containing 10% fetal bovine serum and 1% antibiotics (25.2%). The size distribution of albumin-doxorubicin nanoparticles in cell culture medium (158.23 ± 42.9 nm; PDI: 0.155) demonstrated a similar size distribution to albumin-doxorubicin nanoparticles in PBS (Figure S2). According to these results, albumin-doxorubicin nanoparticles are sufficiently stable against serum proteins. However, compared to the in vitro release profile of doxorubicin in PBS and cell culture medium, doxorubicin was more rapidly released from the albumin-doxorubicin nanoparticles at pH 4.7. 41.5 % of doxorubicin was released at pH 4.7 for 50 h. Regarding to this result, doxorubicin was electrostatically bound to albumin and was pH-dependently released (Figure 2B).

The phospholipid-based microbubbles were filled with a SF_6_ gas core and had size distributions of 1.73 ± 0.34 µm (PDI: 0.297). The albumin-doxorubicin nanoparticles were conjugated onto the surface of the microbubbles and the subsequent size distribution was 2.33 ± 1.34 µm (PDI: 0.395). Following albumin-doxorubicin nanoparticle conjugation, the size distribution of the ADMB complex was slightly larger in comparison to that of the free microbubbles. To increase doxorubicin loading efficiency by raising conjugation ratio of albumin-doxorubicin nanoparticles to microbubbles, albumin-doxorubicin nanoparticles (2 mg of doxorubicin loaded nanoparticle) were conjugated to microbubbles so that the number of microbubbles was 1 × 10^9^. However, the ADMBs were aggregated heterogeneously (Figure S3) Aggressive aggregates of ADMBs were observed by optical microscopy images and the size distribution was incorrectly defined by DLS. This aggregation was induced by numerous reactions between both amine groups on the albumin-doxorubicin nanoparticles and N-hydroxysuccinimide on the microbubble surface. Hence, ADMBs were optimized with a ratio of 1 mg of doxorubicin in albumin-doxorubicin nanoparticles to 1 × 10^9^ microbubbles. In optimized ADMBs, the conjugation of the albumin-doxorubicin nanoparticles to the microbubbles was confirmed by fluorescence emission from the doxorubicin conjugated onto the surface of microbubble without ADMB aggregates (Figure 2C).

### 2.2. Phantom Study for Echogenicity of ADMB Complex

To investigate if the ADMB complex was capable of resonance to ultrasound irradiation for the cavitation effect, the echogenicity was evaluated by visualization with a clinical ultrasound scanner.

**Figure 3:**
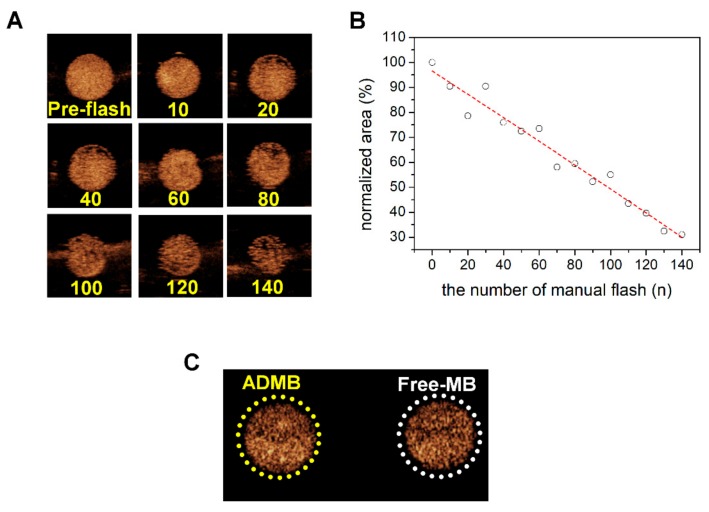
Contrast-enhanced ultrasound images and relative quantification of ultrasound images. (**A**) contrast-enhanced ultrasound images captured at varying numbers of manual flashes. (**B**) Relative quantification of ultrasound image. (**C**) Ultrasound image of the ADMB complex and the free microbubble.

For the ultrasound imaging, 2% of home-made agarose phantom containing 2-holes was used. A contrast-enhanced ultrasound imaging mode demonstrated echogenicity only from the microbubbles. At a low MI of 0.06, the ADMB complexes were stably visualized by microbubble oscillations. However, the echogenicity decreased upon microbubble destruction, depending on the number of manual flashes (mechanical index: 0.68). Manual flashes strengthen the intensity of ultrasound exposure and lead to the destruction of the microbubbles. About 100 times of manual flashing decreased the echogenicity of the microbubbles by about half (55.45%). The echogenicity consistently decreased upon increasing the number of manual flashes (Figure 3A,B). The differences of echogenicity between the free microbubbles and the ADMB complexes were also investigated from the ultrasound images in the same frame, to analyze whether the cavitation effect was altered by conjugation with the doxorubicin-albumin nanoparticles. The echogenicity of the ADMB complexes did not differ from the free microbubbles. The percentages of echogenic area of the ADMB complexes and the free microbubbles were 79.6% and 76.5% respectively, in a relatively equal area (Figure 3C, Table S1).

### 2.3. Enhancement of Cell Uptake and Cell Viability of ADMBs by Sonoporation Effect

**Figure 4:**
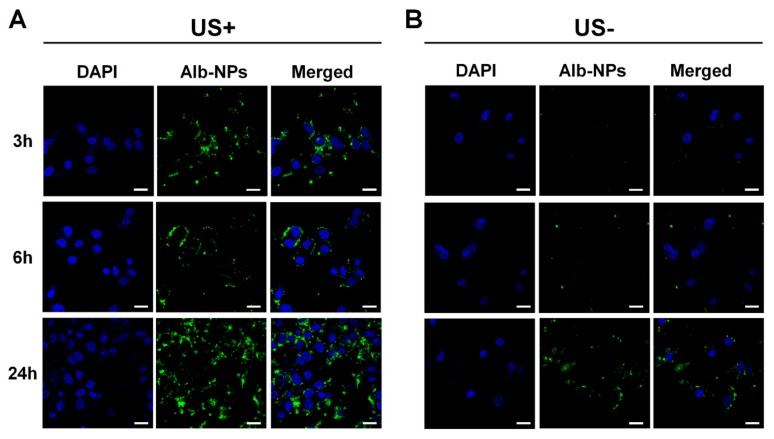
Cellular uptake of albumin nanoparticles after exposure of HepG2 cells to ADMBs. fluorescent image of albumin nanoparticles (**A**) under the ultrasound exposure and (**B**) without ultrasound exposure at 3, 6 and 24 h, respectively (blue; nucleus, green; albumin nanoparticle). Scale bar: 20 µm.

**Figure 5:**
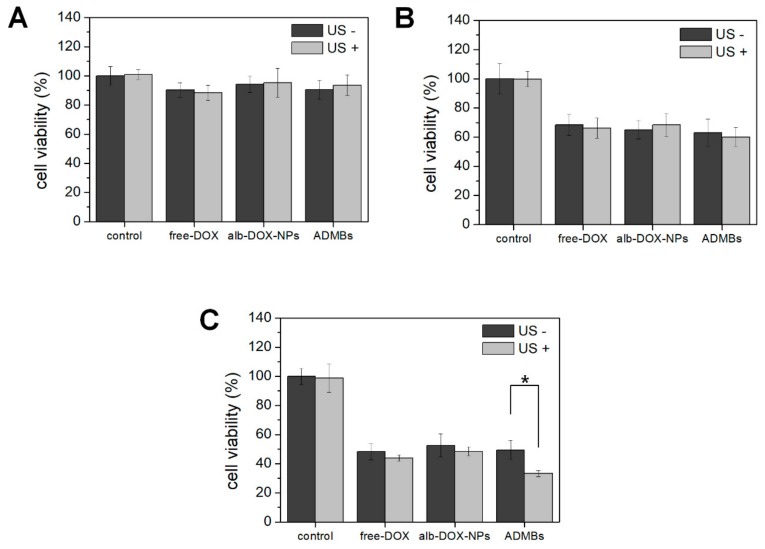
Cytotoxicity of doxorubicin (free-DOX) albumin-doxorubicin nanoparticle (alb-DOX-NPs) and ADMBs at (**a**) 24 h, (**b**) 48 h and (**c**) 72 h with (light gray) or without (dark gray) ultrasound exposure (* *p* < 0.05).

Intracellular uptake and cell viability of ADMB with or without ultrasound exposure were evaluated to investigate the enhancement of the anticancer effect by the sonoporation phenomenon. For tracking nanoparticle uptake into the cell, Alexa555 dye was conjugated to the nanoparticles and the nucleus was stained by DAPI. As shown in Figure 4, albumin nanoparticles were rarely permeable into the cytoplasm within 3 h without ultrasound exposure. A slight fluorescence intensity was detected from nanoparticles at 6 and 24 h post-incubation in the group without ultrasound exposure, because the large size of MBs disturbed the cellular uptake of nanoparticlesr. On the other hands, albumin nanoparticles rapidly penetrated into the cytoplasm within 3 h with the ultrasound exposure. Thus, cellular uptake of albumin nanoparticles was continuously increased time-dependently. At 6 and 24 h post-incubation, albumin nanoparticles were effectively located in the cytoplasm. Regarding these results, the cell viability of ADMBs was evaluated with or without ultrasound exposure. In vitro cell viability was confirmed using a hepatocellular carcinoma cell line (HepG2). To accurately verify that the sonoporation effect between microbubbles and ultrasound is non-toxic, we evaluated the cell viability under various ultrasound exposure conditions with or without microbubbles (Figure S4). Hence, we analyzed cell viability with 1 W/cm^2^ of ultrasound strength and 5% duty cycle (Figure 5). At 24 h post-incubation, the cell viability in all groups was demonstrably similar. However, cell viability was decreased time-dependently after 48 h of incubation. Specifically, ADMBs only demonstrated enhancement of cell viability under the ultrasound exposure after 72 h (*p* < 0.05). As microbubbles resonate with the ultrasound and generated cavitation, albumin-doxorubicin nanoparticles were capable of penetration through the cell membrane more easily than other treatment groups. The treatment groups without microbubbles showed similar levels of cytotoxicity regardless of the ultrasound exposure. In conclusion, cellular uptake and cell viability results demonstrated that ADMBs were capable of not only generating sonoporation effects by cavitation with ultrasound, but also enhancing nanoparticle penetration through the pores in cellular membranes, hence the anticancer effect was enhanced by ADMBs under ultrasound exposure.

### 2.4. Animal Models and Ultrasound Imaging during Treatment Procedure

**Figure 6:**
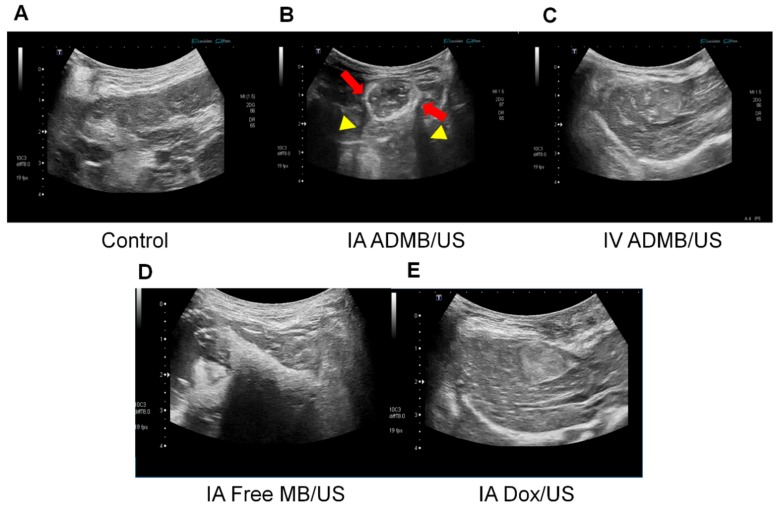
Representative ultrasound images of (**A**) control, (**B**) intraarterial injection of ADMB (IAMB/US), (**C**) intravenous injection of ADMB (IVMB/US), (**D**) intraarterial injection of microbubble (IA Free MB/US) and (**E**) intraarterial doxorubicin (IA Dox/US). Note that strong rim-like enhancement of intratumoral vessels (arrow) with posterior shadow (arrowhead) in IAMB/US group.

A total of 25 VX2 liver tumor rabbit models were created. All rabbits having tumors survived through the tenure of the experiment. The tumors were visualized using ultrasound during the experiments. A strong enhancement of intra-tumoral vessels was clearly demonstrated from the beginning of the injection in the IA-ADMBs and IA-free MB groups (Figure 6B,D). Individuals receiving IV microbubbles (Figure 6C) showed a moderate tumor parenchymal enhancement accompanied by enhanced liver parenchyma and liver vessels. Ultrasound images without microbubbles demonstrated non-enhanced echogenicity (Figure 6A,E) The enhancement of ultrasound echogenicity demonstrated that the delivery efficiency of ADMB in the groups receiving IA microbubbles was higher than that of the groups receiving IV microbubbles. Interestingly, echogenicity of the both IA-ADMBs and IA-free MB demonstrated a similar degree of enhancement. However, the echogenicity enhancement patterns were slightly different. In the US image of ADMBs, increased echogenicity was noted separately at the tumor vessels and tumor parenchyma, whereas the echogenicity of the tumor and adjacent liver tissue in the US image of IA-free MB was enhanced simultaneously. This discrepancy was probably due to the fact that albumin was capable of binding glycoprotein 60 receptor around liver tumor epithelium and of enhancing the anti-cancer effect by targeted delivery [21].

### 2.5. Antitumor Efficacy of Albumin-Doxorubicin Nanoparticle-MB Complex by Quantitative MR Imaging and Pathology

**Figure 7:**
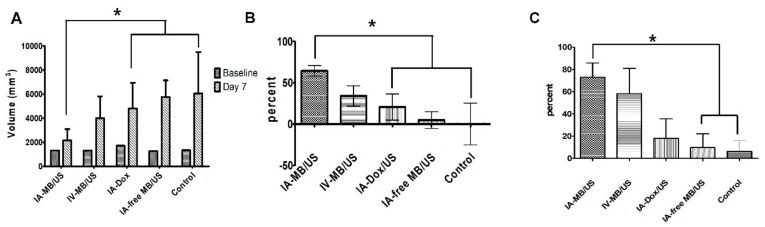
Quantitative volumetric image analysis at baseline and on day 7 after treatment, in IA ADMB/US, IV ADMB/US, IA Doxorubicin/US, IA free MB/US, and the untreated control groups. (**A**) Change in tumor volumes. (**B**) The Volume Inhibition Rate (VIR) of each group. (**C**) Percent change in ADC values across the experimental period among the groups. Each bar represents mean SD, * *p* < 0.05 versus IA Doxorubicin/US, IA free MB/US, and untreated control group.

**Figure 8:**
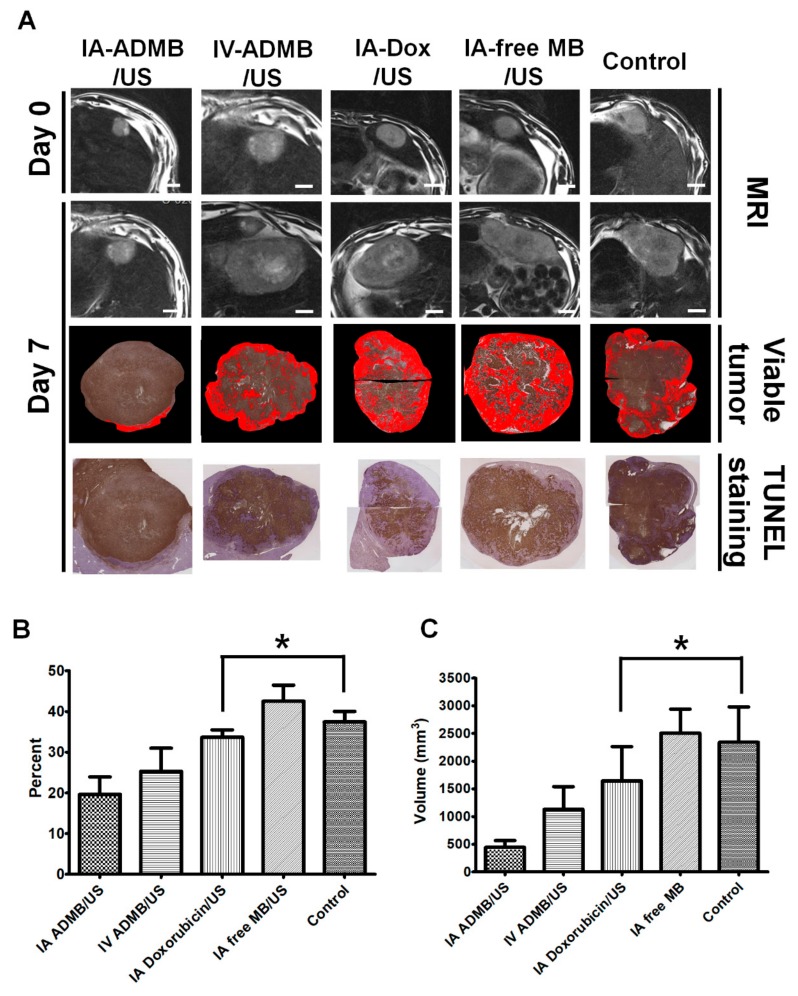
Representative MRI and histo-segmentation images of each group, Scale bar: 10 mm (**A**). Quantitative analysis of viable tumor fraction (**B**). Estimated viable tumor volume (**C**) at day 7 after treatment. Red area represents the viable portion of tumor; each bar represents mean SEM., * *p* < 0.05 versus IA doxorubicin/US, IA free MB/US, and untreated control group.

The anticancer efficacy of ADMB was evaluated using quantitative MR imaging (Figure 8A). The measured mean tumor volume at baseline and at 7 days after drug administration, volume inhibition rate (VIR), and change in ADC values are summarized in Figure 7. There was no significant difference in tumor size among groups at baseline (*p* = 0.614). The mean tumor volume of IA-ADMB.US (2156.57 ± 849.86 mm^3^) was significantly small compared to that of IV-ADMB/US (3777.47 ± 1950.72 mm^3^). The mean tumor volume of IV-ADMB/US was larger than that of IA-ADMB/US. And the tumor volume of IA-Dox/US, IA-free MB/US and control were 4811.02 ± 2132.69 mm^3^, 5770.06 ± 1382.78 mm^3^, and 6063.97 ± 3432.51 mm^3^, respectively. Comparison of tumor volume IA-Dox/US, IA-free MB/US and control was not statistically significant. Among the five groups, IA-ADMB/US achieved a maximal reduction in tumor volume of 64.44 ± 15.35% on day 7 as indicated by the MRI (Table 1). Both the IA-ADMB/US and IV-ADMB/US showed an increase in the ADC values after treatment by more than 50%, compared to pre-treatment values, which suggested the loss of diffusion-restrictive lesions such as tumor parenchyma. For group of IA-ADMB/US, the VIR and percent change in ADC value were significantly higher than others (Figure 7). In addition, the VIR and percent change in ADC value in IV-ADMB/US tended to be lower than the corresponding values of group IA-ADMB/US and higher than the corresponding values of IA-Dox/US, IA-free MB/US and control. However, the results were not statistically significant. The histologically viable tumor percentage was quantified using a slide-by-slide segmentation of the H&E staining images and TUNEL-stained images that were generated to investigate the entire section of the whole tumors. The pathological analysis performed on day 7 showed low viable tumors in IA-ADMB/US (19.60 ± 12.1%, 25.29 ± 14.00% and 33.65 ± 4.09%, 42.48 ± 8.85%, 37.42 ± 5.80% for IA-ADMB/US, IV-ADMB/US, IA- Dox/US, IA-free MB/US and control, respectively; Figure 8A,B, Figure S5 and Table 2). IA-ADMB/US demonstrated a significantly higher necrotic fraction and a lower estimated viable tumor volume, compared to IA- Dox/US, IA-free MB/US and control (Figure 8B,C). Similar to the MR-based analysis, the viable tumor percentage and the estimated viable tumor volume of IV-ADMB/US tended to be higher than the corresponding values of IA-ADMB/US and lower than the corresponding values of others; the difference, however, was not statistically significant.

### 2.6. Biochemical Liver Toxicity Evaluation

**Figure 9:**
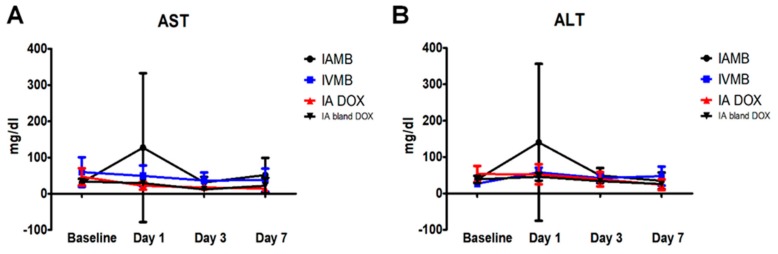
Graphs depicting liver enzyme values (AST and ALT) changes over the observation period for each treatment group. (**A**) Graph depicting the plasma concentration changes in aspartate transaminase (AST) over the 7-day observation period (expressed in mg/dl). (**B**) Graph depicting the plasma concentration changes in alanine transaminase (ALT) over the 7-day observation period. No significant changes in AST and ALT levels at each time interval throughout the observation period among the five groups.

All the animals showed a tendency to reach the highest values of AST and ALT enzymes at 24 h after treatment, which gradually decreased and returned to baseline values at 7 days after treatment. The AST and ALT values, noted at specific intervals of time starting from baseline to the end of the observation period, did not differ significantly among the five groups (Figure 9).

## 3. Discussion

In this study, we prepared a novel drug delivery system with a dual function, acting as a drug carrier and an ultrasound contrast, by combining the advantages of microbubbles and bio-compatible nanoparticles. Albumin-doxorubicin nanoparticles were synthesized by a desolvation technique. The doxorubicin was strongly bound to the albumin and the correlation coefficient between doxorubicin and albumin was 0.98 [22]. The albumin-doxorubicin complexes were conjugated to the nanoparticles by cross-links between the amine group of albumins and aldehyde groups [23]. Being covalent in nature, these bonds were extremely strong. The doxorubicin molecules were stably loaded by both surface absorption and incorporation into the nanoparticles [22,24]. This structure is advantageous since the nanoparticles are not easily degraded and the doxorubicin incorporated within the nanoparticle is safely protected from different enzymes in the blood vessels. In addition, as shown in Figure 2B and Figure S2, doxorubicin was released in a sustained manner for a long period of time owing to the solid structure [25]. The conjugation of albumin-doxorubicin nanoparticles onto the surface of the microbubbles was also based on the amide bonding previously described. This amide bond forms between the numerous primary amines of albumin and N-hydroxysuccinimide of the microbubbles [18]. This rapid and strong bond is also easily induced within 1 h. The albumin-doxorubicin nanoparticles do not easily extravasate owing to the large size of the microbubbles, and are capable of safely delivering doxorubicin to the target site without mid-way losses, until the target site is exposed to ultrasound irradiation.

To confirm that the ADMBs resonate to ultrasound, we investigated the echogenicity of ADMBs using a commercial ultrasound scanner, which is equipped with a transducer having a frequency range of 2–9 MHz. In an in vitro phantom experiment, microbubbles were continuously visualized during ultrasound irradiation at a low MI of 0.06. The results showed that the microbubbles oscillated and generated acoustic wave pressure necessary for a stable cavitation. However, at a MI of 0.06, this behavior is not enough to generate sonoporation, because a MI of 0.06 is sufficiently low and this MI value is only for adaptation to diagnosis by ultrasound. However, collapse of microbubbles was demonstrated under the ultrasound radiation with MI 0.68 by manual flash. The destruction of microbubbles by the application of strong, intense ultrasound irradiation leads to an asymmetric gas infusion with high pressure from the core, which induces a temporary stress on the cellular membrane. This phenomenon is known as inertial cavitation and our ADMB complex was proven to induce inertial cavitation, as depicted in Figure 3. In addition, the driving frequency for inertial cavitation was 2–9 MHz, and this is the frequency which is generally applied to abdominal organs in the clinical field. Theoretically, the penetration depth and frequency of ultrasound are critically related. A lower frequency permeates more to deeper regions, whereas the resolution of ultrasound imaging is clear in the diagnostic field. However, in clinical research, studies generally use low frequency ultrasounds for a deeper penetration and high intensity ultrasounds for an enhanced ultrasound trigger. In our previous study, we measured that the optimal resonance frequency of the albumin nanoparticle-conjugated microbubble is 3 MHz [18]. Therefore, a resonance frequency of 2–9 MHz was optimum for both our ADMBs and the hepatocellular carcinoma. We also analyzed the influence of albumin-doxorubicin nanoparticle conjugation on the echogenicity. As conjugation induces changes in the microbubble surface, corresponding changes in shell elasticity and stiffness are possible [26,27]. It is perhaps this change which is able to reduce the echogenicity. However, as shown in Figure 3C, the echogenicity was rarely influenced.

Regarding these characteristics of ADMBs, intracellular uptake and anticancer effect was evaluated, in order to study the generation of sonoporation effects by ADMBS and to enhance the anticancer effect. Cytotoxicity of ADMBs was enhanced under ultrasound, whereas the cytotoxicity of doxorubicin or albumin-doxorubicin was not related to ultrasound exposure. Theoretically, a high intensity of ultrasound such as HIFU can generate sonoporation and enhance intracellular delivery. However, high intensity of ultrasound to open cellular membranes usually stresses the surroundings and occasionally induces cytotoxicity to the normal cells or organs. Therefore, microbubbles were sufficiently beneficial for the generation of sonoporation effects with lower ultrasound pressure and enhancement of drug delivery into the cytoplasm. In our study we proved, as shown in Figure 4 and Figure 5, that intracellular delivery and anticancer effects in the presence of microbubbles under ultrasound were enhanced within 3 h, whereas ADMBs without ultrasound exposure did not penetrate in 6 h, because sonoporation was not induced and large size of microbubbles interfered with penetration through the cell membrane. Therefore, the cytotoxicity of ADMBs under the ultrasound exposure was strongest, compared to doxorubicin and albumin-doxorubicin nanoparticles.

The second section of our study comprehensively evaluated the in vivo antitumor effect of ADMB following ultrasound activation. As we had expected, the combined therapy of IA ADMB/US administration led to the highest reduction in tumor volume. This was also supported by the fact that IA-ADMB/US group showed the maximal increase in ADC values post-treatment in MR, the highest necrotic fraction and the lowest viable tumor volume revealed by histosegmentation analyses. Moreover, pathological analysis revealed intense and homogeneous necrosis throughout the whole tumor section in the IA-ADMB/US group and viable tumors were nearly absent on the tumor periphery. In contrast, tumor necrosis in the untreated control group was focal and heterogeneous, and there were abundant finger-like viable tumor portions in the periphery. It is presumed that the IA route delivered a high concentration of ADMB which resulted in maximal cavitation and sonoporation in the target tumor site, causing enhanced drug penetration and cytotoxicity to tumor tissues. To our knowledge, this study a first of its kind to investigate and report the promise of IA delivery of ADMB following ultrasound activation in an orthotropic liver tumor animal model larger than rodents. Interestingly, tumor suppression in the IV ADMB/US group was modest but not statistically significant to other groups. Unlike previous studies which primarily used small animals [28,29,30], our study used rabbits, which are relatively large animals with more blood volumes, and have longer distances between tumors and ultrasound probes. It is perhaps these differences which resulted in the diverse treatment outcomes in the IV ADMB/US group. Previous studies [18,19,28,31,32,33] used high-intensity, low-intensity focused ultrasound or self-made ultrasound transducers to elucidate the feasibility of sonochemotherapy. However, these probes are not generally used in diagnostic imaging, and therefore cannot be applied for the same. In our study, we used an ultrasound scanner used for clinical purposes to simultaneously visualize the tissues of the target tumor and to deliver ultrasound irradiation for activating microbubble-assisted drug delivery. This allows the advantage of directly monitoring drug delivery while treating the target tumor, which means that it can be easily applied to clinical studies in the future. It is important to evaluate the safety profile while investigating new drug delivery systems. In our study, there were no significant differences in the liver enzyme levels analyzed at specific time intervals, between the IA ADMB/US group and other groups. In addition, no animal deaths occurred due to complications arising from microbubble injection. This indicates that the IA ADMB/US therapy could be effective and safe for the treatment of liver cancers.

We acknowledge several limitations of our study. First, we did not study the treatment outcomes when only ADMB was administered without ultrasound irradiation. However, the efficacy of drug-loaded microbubbles in combination with microbubble destruction is a well-known drug delivery system, as many previous reports suggest [18,28,31,32,33,34,35,36]. Second, the concentration of doxorubicin in tumors was not directly measured. However, the IA ADMB/US group showed excellent tumor suppression as observed by both volumetric and quantitative analyses, indicated by changes in the ADC values, MR and necrotic fraction, and viable tumor volume. Moreover, the free MB/US group did not show significant tumor inhibition compared to the control group. These findings indirectly support that the enhanced anticancer effect in the IA ADMB/US group was caused by the application of an improved drug delivery system.

## 4. Materials and Methods

### 4.1. Materials

1,2-Distearoyl-sn-glycero-3-phosphocholine (DSPC) was purchased from NOF Corporation (Tokyo, Japan). 1,2-Distearoyl-sn-glycero-3-phosphoethanolamine-N-[succinyl(polyethylene glycol)-2000] (DSPE-PEG2k-NHS) was purchased from Nanocs Incorporated (Boston, MA, USA). Human serum albumin, 8%-glutaraldehyde, and 99%-ethanol were purchased from Sigma-Aldrich (St. Louis, MO, USA). Doxorubicin was purchased from the Il-Dong Pharmaceutical Company (Seoul, Korea). Microcatheters (Progreat 2.0F, Terumo, Japan) were obtained from the Terumo Korea Corporation (Seoul, Korea). The animals were purchased from Orient Bio Co. (Seongnam, Korea). All the other chemicals and solvents were of analytical grade.

### 4.2. Preparation of ADMB Complex

The ADMB complex consisted of two main parts: the albumin nanoparticle loaded with doxorubicin and the phospholipid-based microbubble; the complex was fabricated as per the sequence of procedures mentioned hereafter. First, the albumin nanoparticle was fabricated by a desolvation method; 150 mg of human serum albumin and 5 mg of doxorubicin were dissolved in water and the pH was adjusted to 8.5 by using 1 M NaOH. After stirring (at 600 rpm) for 2 h, 8 mL of ethanol (99.9%) was continuously added in a dropwise manner for transforming the mixture into doxorubicin-albumin nanoparticles, indicated by the development of turbidity in the mixture; 100 µL of 8%-glutaraldehyde was added cross-linking the nanoparticles. After stirring overnight, the albumin-doxorubicin nanoparticles were purified by centrifugation for 10 min at 4 °C at 15,000 rpm, and were resuspended in equal volume of 0.01 M phosphate buffer saline (pH 7.4). The loading efficiency of doxorubicin was calculated by analyzing the quantity of unloaded doxorubicin in the supernatant after centrifugation. The amount of doxorubicin in the supernatant was measured by a UV-Vis spectrometer.

DSPC and DSPE-PEG2k-NHS dissolved in chloroform in a 9:1 molar ratio. The chloroform was fully evaporated for fabrication of a thin phospholipid film. This thin phospholipid film was hydrated by 0.01 M PBS at a temperature above the phase transition temperature of DSPC (55 °C). A 2 mL vial was filled with the volume of phospholipid solution (at a concentration of 0.5 mg/mL) and sulfur hexafluoride gas (SF_6_) at the headspace. Sequentially, the microbubbles were formulated by activation with Vialmix^TM^ for 45 s.

The albumin-doxorubicin nanoparticles were conjugated with the microbubbles by adding the albumin-doxorubicin nanoparticles to the microbubbles at a concentration of 1 mg/mL. The albumin-doxorubicin nanoparticles were conjugated to the microbubbles via amide bonds between the primary amine of the nanoparticles and the NHS from the microbubble surface. After 1 h for conjugation of albumin-doxorubicin to surface of NHS functionalize microbubble, centrifugation at 3000 rpm for 5 min was performed at three times for purification of ADMBs. Unbound albumin-doxorubicin was removed and ADMBs were re-suspended by 0.01 M PBS.

### 4.3. In Vitro Release Test for the ADMB Complex

In order to investigate doxorubicin release from the ADMB complex, the complex was sealed with a 3000 Da (MW) cut-off dialysis membrane. The ADMBs loading 1 mg of doxorubicin in the dialysis membrane was placed into the tube filled with 10 mL of phosphate buffer saline, saline with adjustment to pH 4.7 or cell culture medium containing 10% of fetal bovine serum and 1% of antibiotics. This ADMBS was stored at 37 °C and 500 rpm of shakes (*n* = 3). The released doxorubicin was measured by a UV-Vis spectrometer.

### 4.4. Phantom Study for Echogenicity of the ADMB Complex

The echogenicity of the ADMB complex was evaluated using a commercial ultrasound scanner equipped with a transducer having a frequency range of 2–9 MHz. For the ultrasound imaging, home-made agarose phantoms containing two holes of 2 cm depth was used. The holes in the agarose phantoms were filled with the ADMB complex at the concentration of 5 µg/mL and degassed water, respectively. For studying microbubble stability, ultrasound imaging was performed at a Mechanical Index (MI) of 0.06 and manual flash mode (MI:0.68) was performed with 3 s time interval for microbubble destruction (iu-22 Philips Medical System, Philips, Bothell, WA, USA). The stability of the ADMB complex was analyzed by decreasing the ratio of echogenic area (pixel) with the ImageJ software (version 1.45 s; National Institutes of Health, Bethesda, MD, USA).

### 4.5. Cellular Uptake and Cell Viability of ADMB under the Ultrasound Exposure

To determine that ADMB enhances cell permeability under the ultrasound exposure, hepatic cellular carcinoma cell line, HepG2, was applied. HepG2 cells (5 × 10^4^) were seed to the 8 well cell culture chamber and incubated for overnight. To fluorescently visualize, fluorescence dye, NHS-alexa488, was labeled to human serum albumin nanoparticle without doxorubicin loadings. Florescence dye was conjugated by amide bond between albumin nanoparticle and NHS. Fluorescently labeled ADMBs were treated and were exposed to the ultrasound wave (Sonidel SP100 sonoporator, Sonidel Ltd., Dublin, Ireland). Ultrasound was irradiated to each well with the 1 W/cm^2^ of strength and 5% of duty cycle for the 1 min. At the post-incubation of 3, 6 and 24, HepG2 cells were washed three times and fixed by 4%-paraformaldehyde. And nucleus was stained by DAPI. Cellular uptake of ADMBs were observed by confocal laser microscopy.

Cell viability of ADMBs under the ultrasound exposure was measured by MTT assay. HepG2 cells (1 × 10^4^) were seeded in each well of 96 well plate. After overnight for incubation, doxorubicin, free-microbubble, doxorubicin loaded albumin nanoparticles or ADMBs were treated to each well. The mass of doxorubicin in each well was equalized to 5 µg. Ultrasound was irradiated with the 1 W/cm^2^ of strength and 5% of duty cycle for 30 s. And each well was washed 3 times at the post incubation of 3 h. And cell viability after 24, 48, 72 h was measured at the wavelength of 540 nm by ELIZA reader.

### 4.6. Animal Model Preparation

The animal research protocols followed in this study were approved by Seoul National University of medicine institutional Animal Care and Use Committee. Twenty-five male New Zealand white rabbits weighing between 3000 and 3500× *g* were used for our study. The animals were housed in cages with a 12-hour light/dark cycle and ad libitum access to standard rabbit chow diet and water. During all procedures, the animals were anesthetized with intramuscular injections of 5 mg/kg body weight of tiletamine-zolazepam (Zoletil 50; Virbac, Carros, France) and a 2 mg/kg body weight of 2% xylazine hydrochloride (Rompun; Bayer, Seoul, Korea). The VX2 carcinoma strain was maintained in the right hind limb of a carrier rabbit through deep intramuscular injection throughout the study. Briefly, the left lobe of the animal’s liver was exposed surgically and a small piece of tumor tissue (1 mm^3^) freshly harvested from the maintained tumor was directly implanted at the subcapsular area of the liver for each rabbit, as described in previous studies [10,37,38]. The tumor was incubated for 17–18 days.

### 4.7. MR Imaging

All animals underwent MR imaging at day 0 (baseline before treatment) and at day 7 following IA or IV infusion in the groups A to D which received treatment, and the untreated group E. A 3.0-T clinical MR scanner (TimTrio; Siemens Healthcare, Erlangen, Germany) with a knee coil was used to improve SNR and spatial resolution. The animals were fixed on a board in a supine position, and an abdominal bandage was tightly applied to reduce any movement artifact. Axial T_2_-weighted turbo spin-echo (repetition time/echo time: 4100 milliseconds/150 milliseconds; echo train length: 14; section thickness: 3 mm; field of view: 130 × 130 mm, matrix: 512 × 358; number of excitations: 2.0) and IVIM Diffusion-weighted image (free breathing single-shot echo-planar imaging pulse sequence with diffusion gradients applied in three orthogonal directions: 2700/63; section thickness: 3 mm; number of sections: 20; number of signals acquired: 8; field of view: 14 × 14 cm^2^; matrix: 128 × 128; and four b values (0, 15, 200, and 800 s/mm^2^)) were acquired [39]. The images were evaluated using a dedicated workstation for picture archiving and communication system (m-view; Marotech, Seoul, Korea).

### 4.8. Grouping and Drug Delivery

The study design is summarized in Figure S1. On the basis of the treatment procedure, the animals were divided into five groups having similar tumor volumes: animals receiving an intra-arterial (IA) infusion of ADMB/US (group IA-ADMB/US, *n* = 6), animals receiving an intravenous (IV) infusion of ADMB/US (group IV-ADMB/US, *n* = 6), animals receiving an IA infusion of free MB/US (group IA-free MB/US, *n* = 5), animals receiving an IA infusion of doxorubicin/US (group IA- Dox/US, *n* = 3), and the untreated control (*n* = 5). The dose of doxorubicin delivered was 1 mg for all groups except for groups of IA-free MB/US and control. In each group except the control, an infusion pump (Genie plus, Kent Scientific Corporation, Torrington, CT, USA) was used. IA-ADMB/US underwent an IA delivery of ADMB in 3 mL of Iopamidol (Pamiray^®^, Seoul, Korea) contrast media via the proper hepatic artery. ADMB was administered in group of IV-ADMB/US via the left marginal ear vein. In IA-free MB/US, the microbubble-contrast solution without doxorubicin was administered via the proper hepatic artery under the same conditions as group of IA-ADMB/US. The number of microbubbles in group IA-and IV-ADMB/US and IA-free MB/US were 9 × 10^8^ in 3 mL of Iopamidol, respectively. Group of IA- Dox/US received a mixture of 1 mg of doxorubicin in 3 mL of contrast media via the proper hepatic artery. All the injections for groups were administered at a rate of 1 mL/min for 3 min, using the infusion pump for an accurate and homogenous infusion.

For the IA delivery, an 18-gauge catheter (BD Angiocath Plus with intravenous catheter, Becton-Dickinson, Korea) was inserted into the right central auricular artery for arterial access. To reach the proper hepatic artery, a 2.0-Fr microcatheter (Progreat; Terumo, Tokyo, Japan) was advanced via the catheter into the descending aorta [37,40]. After performing hepatic arteriography to confirm tumor staining and following visualization of the proper hepatic artery, the microcatheter was advanced selectively until the catheter tip was gently positioned at the proximal portion of the proper hepatic artery. The solution prepared for each group was then administered using an infusion pump (Genie plus, Kent Scientific Corporation) through the microcatheter at a rate of 1 mL/min for 3 min, to avoid reflux of the injected complex from the proper hepatic artery [31,37]. To access the systemic venous system, an 18-gauge catheter was inserted into the left marginal ear vein. The pressure line was then connected to catheter, and the solution was infused similarly. When the solution was completely injected, the microcatheter was removed, and the puncture site was compressed carefully to achieve hemostasis.

### 4.9. Ultrasound and Microbubble Activation

The abdominal hairs of the rabbits were carefully removed just prior to ultrasonography. The ultrasonography was performed by a radiologist both before and during drug administration in groups IA-ADMB/US, IV-ADMB/US, IA- Dox/US and IA-free MB/US using the Aplio 500 ultrasonographic system (Toshiba Medical Systems, Otawara, Japan), with 674 BT with an 8 MHz center frequency convex transducer. A fundamental B-mode ultrasound (a dynamic range of 65; a mechanical index of 1.5; a gain of 90; and a depth of 4 cm) was used to detect the VX2 tumors. After localization and a morphological examination of the tumor, the optimal plane was determined and the skin was marked. The vascular recognition imaging mode with a low MI of 0.06 was used to detect signals generated by the microbubbles. As soon as an IA or IV delivery of the mixture through the infusion pump began, the vascular recognition mode was used to confirm the presence of tumor enhancement. Simultaneously, using a continuous up-and-down sweeping of the probe at the skin marking site, the B-mode was used to irradiate ultrasound energy to the tumor for 3 min (J.H.L). After cessation of infusion, additional ultrasound irradiation was applied for 5 min for activating the microbubbles, resulting in a total of 8 min of bubble activation for each rabbit using the B-mode ultrasound.

### 4.10. Imaging Analysis

A radiologist who was blind to the information regarding the experimental group evaluated the MR images on a picture archiving and communications system workstation. The T_2_-weighted images were used to confirm tumor formation and to measure the maximal longitudinal diameter (length) and maximal transverse diameter (width) of the tumors. Tumor volume was calculated from the measurements determined by MR imaging, using the modified ellipsoidal formula, tumor volume = 1/2(length × width^2^) [34,38]. The volume inhibition rate (VIR) of tumor growth was calculated using the formula IR = (T_c_ − T_t_)/T_c_ × 100%, where T_c_ represented the tumor volume of group E (control group) and Tt represents the tumor volume of each treatment group. The value of the Apparent Diffusion Coefficient (ADC) was measured quantitatively using the largest cross-section of the tumor visualized on the ADC map. The changes in the ADC values before and after TACE were evaluated.

### 4.11. Pathological Analysis

On day 7, all animals were pre-anesthetized and sacrificed with an intravenous injection of xylazine hydrochloride, and the whole tumor was harvested after follow-up imaging. Each tumor was fixed in 10% buffered formalin. The specimen was then embedded in paraffin, cut into 4 µm sections, and the largest cross-section of the tumor was stained with hematoxylin and eosin for basic histopathological examinations. The section was consecutively treated with terminal deoxynucleotidyl transferase dUTP nick end labeling (TUNEL) staining (ApopTag^®^ Peroxidase in situ Apoptosis Detection Kit, Merck KGaA, Darmstadt, Germany) for evaluating tumor viability. After digital images of the histology slides were obtained (Leica Microsystems, Mannheim, Germany), the viable tumor percentage per tumor was calculated using image analysis software (ImageJ, version 1.45 s; National Institutes of Health, Bethesda, MD, USA). In brief, the viable portion of each TUNEL stained image of the whole tumor was measured by threshold intensity. Then, percentage of viable tumor region was calculated by ratio between whole tumor area and viable tumor region. This analysis was performed by an experienced radiologist who was blind to all experimental data, in order to ensure concordance. The estimated viable tumor volume after treatment was calculated as follows: Calculated tumor volume on day 7× viable percentage of the tumor.

### 4.12. Biochemical Liver Toxicity Assessment

Blood samples for assessing liver toxicity were gathered at baseline and at 1-, 3-, and 7-day intervals after treatment. Liver function tests included the assessment of liver enzymes (aspartate transaminase (AST) and alanine transaminase (ALT)).

### 4.13. Statistical Analysis

All the data in the study are reported as the mean ± SD. The nonparametric analysis was conducted using the Kruskal-Wallis test to compare the tumor volume, volume inhibition rate, changes in ADC value, tumor viability, and estimated viable tumor volume in the 5 experimental groups. When positive results were encountered, the Mann-Whitney post-hoc test was used for one-to-one group comparisons. Data processing and analysis were performed using the Statistical Package for the Social Sciences version 18.0 (SPSS Inc, IBM, Chicago, IL, USA). A two-sided *p*-value of less than 0.05 indicated that the groups differed significantly in terms of statistical results.

## 5. Conclusions

In the present study, the ADMB complex was developed for enhancing the therapeutic efficiency of drugs used in hepatocellular carcinoma. This complex can resonate to ultrasound irradiation and induce sonoporation. Using orthotopic experiments, we proved the anticancer effect of this treatment strategy using the VX2 rabbit tumor model. Our strategy is more effective than existing systems because it: (1) has an enhanced loading efficiency; (2) is optimized to resonate at ultrasound frequencies of 2–9 MHz, making it ideal for the treatment of hepatocellular carcinomas, and (3) induces a more effective anticancer effect when combined with an intra-arterial administration route in rabbits. In conclusion, the IA administration of ADMB followed by microbubble activation using a clinical ultrasound probe can take advantage of simultaneously monitoring drug delivery while treating the target, to achieve a better antitumor effect. This novel drug delivery system may help to effectively deliver chemotherapeutics to liver tumors and warrants further investigation for the treatment of advanced liver cancers.

## Figures and Tables

**Table 1:** The tumor volume and volume inhibition rate (VIR) of each group.

Group	Tumor Volume (mm^3^) at Baseline	Tumor Volume (mm^3^) at 7 Days after Delivery	VIR (%)
Control	1331.21 ± 481.67	6063.97 ± 3432.51	-
IA-free MB/US	1266.54 ± 527.06	5770.06 ± 1382.78	4.85 ± 22.80
IA-Dox/US	1712.71 ± 431.79	4811.02 ± 2132.69	20.66 ± 35.16
IV-ADMB/US	1245.30 ± 811.94	3777.47 ± 1950.72	34.10 ± 30.09 *
IA-ADMB/US	1313.08 ± 740.77	2156.57 ± 849.86	64.44 ± 15.35 *^,^**

* *p* < 0.05 compared with the control group; ** *p* < 0.05 compared with IA-Dox/US and IA-free MB/US groups.

**Table 2:** The viable tumor percentage and estimated viable tumor volume of each group.

Group	Viable Tumor (%)	Estimated Viable Tumor Volume (mm^3^)
Control	37.42 ± 5.80	2339.55 ± 1433.18
IA-free MB/US	42.48 ± 8.85	2503.82 ± 975.32
IA-Dox/US	33.65 ± 4.09	1526.89 ± 731.75
IV-ADMB/US	25.29 ± 14.00	1130.98 ± 1003.78
IA-ADMB/US	19.60 ± 10.55 *^,^**	429.0 ± 291.09 *^,^**

* *p* < 0.05 compared with the control group. ** *p* < 0.05 compared with IA Dox/US and IA free MB/US groups.

## Supplementary Materials

The following are available online at https://www.mdpi.com/2072-6694/11/4/581/s1, Figure S1: Study design of the VX2 rabbit liver tumor treatment protocol, Figure S2: Size distribution of albumin-doxorubicin nanoparticle in DMEM containing 10% fetal bovine serum and 1% antibiotics after 1 day, Figure S3: Heterogenous ADMBs, Figure S4: Cell viability under the various conditions of ultrasound exposure and microbubble, Figure S5:TUNEL assay images of tumor region in (A) IA-ADMBs, (B)IV-ADMBs (C) blend microbubble, Table S1: Percentage of echogenic area depending on the number of manual flashes.
