# Supplementary material for: Antitumor Effects of Intra-Arterial Delivery of Albumin-Doxorubicin Nanoparticle Conjugated Microbubbles Combined with Ultrasound-Targeted Microbubble Activation on VX2 Rabbit Liver Tumors
Source: Cancers (Basel). 2019 Apr 24;11(4):581. doi: 10.3390/cancers11040581 (PMC6521081; doi:10.3390/cancers11040581)
Supplement: Supplementary file 1 [file cancers-11-00581-s001.pdf]

Supplementary Materials: Antitumor Effects of Intra-Arterial Delivery of Albumin-Doxorubicin Nanoparticle Conjugated Microbubbles Combined with Ultrasound-Targeted Microbubble Activation on VX2 Rabbit Liver Tumors

Jae Hwan Lee, Hyungwon Moon, Hyounkoo Han, In Joon Lee, Doyeon Kim, Hak Jong Lee, Shin-Woo Ha, Hyuncheol Kim and Jin Wook Chung

Table S1. Percentage of echogenic area depending on the number of manual flashes.

Number of Manual Flashes (n)	0	10	20	40	60	80	100	120	140
Normalized Echogenic Area (%)	100	90.41	78.54	75.93	73.43	59.45	55.03	39.60	30.96

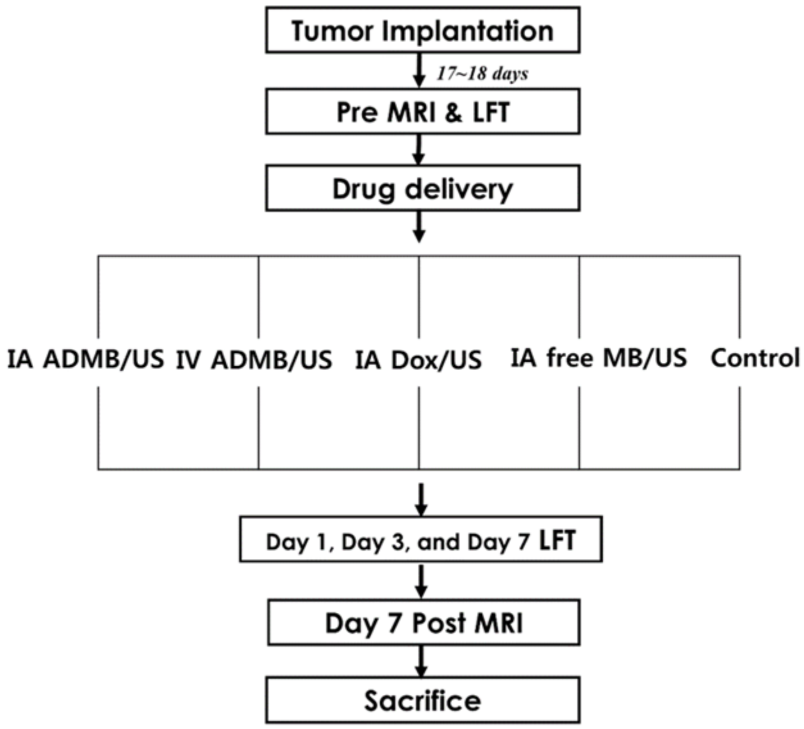

Figure S1. Study design of the VX2 rabbit liver tumor treatment protocol.

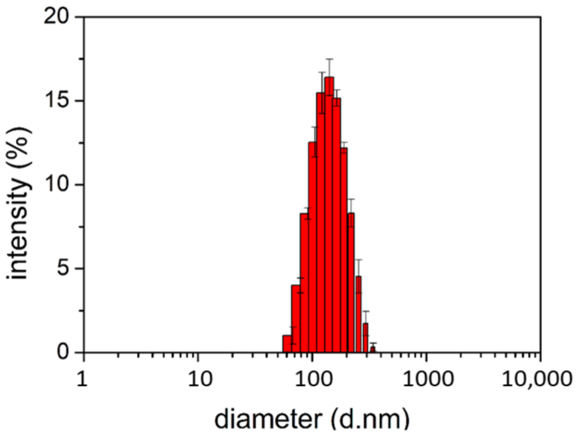

Figure S2. Size distribution of albumin-doxorubicin nanoparticle in DMEM containing 10% fetal bovine serum and 1% antibiotics after 1 day.

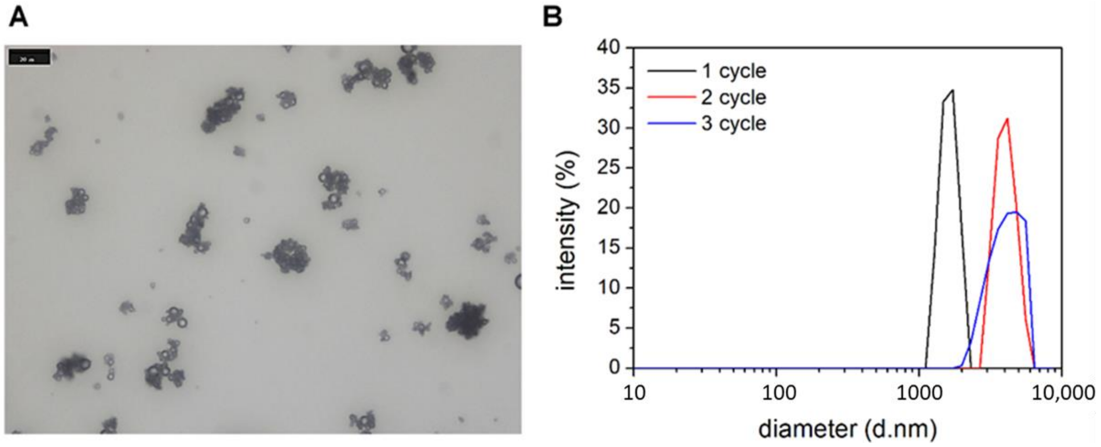

Figure S3. Heterogenous ADMBs. (**A**) optical image and (**B**) size distribution. (scale bar: 10 μm).

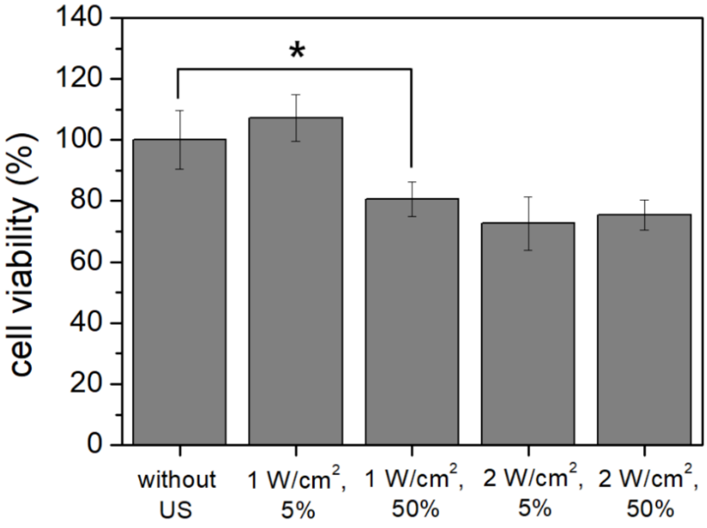

Figure S4. Cell viability under the various conditions of ultrasound exposure and microbubble. ultrasound exposure with 1W/cm2 and duty 5% is only non-toxic condition. Other ultrasound conditions decreased cell viability. (* $p < 0.05$)

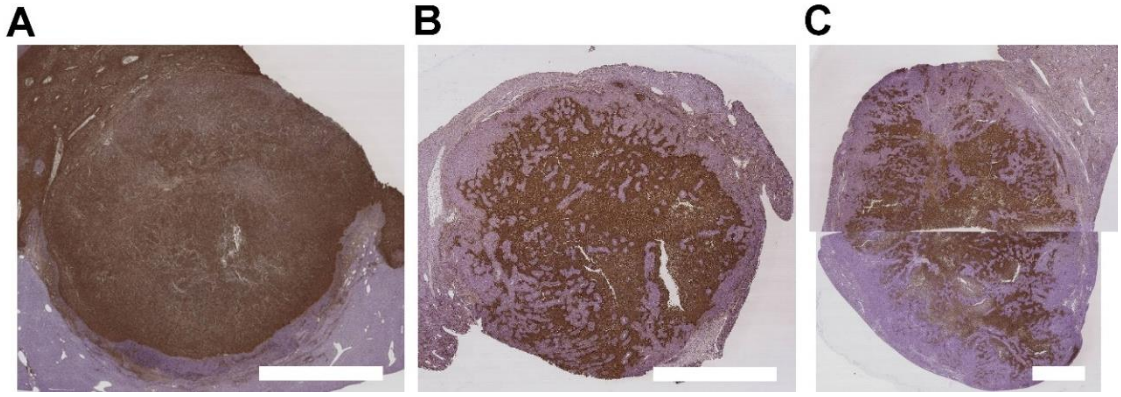

Figure S5. TUNEL assay staining images of tumor region in (**A**) IA-ADMBs, (**B**)IV-ADMBs (**C**) blend microbubble. (Scale bar: 5 mm).

© 2019 by the authors. Licensee MDPI, Basel, Switzerland. This article is an open access article distributed under the terms and conditions of the Creative Commons Attribution (CC BY) license (http://creativecommons.org/licenses/by/4.0/).
